# Image denoising and segmentation model construction based on IWOA-PCNN

**DOI:** 10.1038/s41598-023-47089-6

**Published:** 2023-11-13

**Authors:** Xiaojun Zhang

**Affiliations:** College of Software Technology, Henan Finance University, Zhengzhou, 450000 China

**Keywords:** Applied mathematics, Computer science

## Abstract

The research suggests a method to improve the present pulse coupled neural network (PCNN), which has a complex structure and unsatisfactory performance in image denoising and image segmentation. Then, a multi strategy collaborative improvement whale optimization algorithm (WOA) is proposed, and an improved whale optimization algorithm (IWOA) is constructed. IWOA is used to find the optimal parameter values of PCNN to optimize PCNN. By combining the aforementioned components, the IWOA-PCNN model had the best image denoising performance, and the produced images were crisper and preserve more information. IWOA-PCNN processed pictures have an average PSNR of 35.87 and an average MSE of 0.24. The average processing time for photos with noise is typically 24.80 s, which is 7.30 s and 7.76 s faster than the WTGAN and IGA-NLM models, respectively. Additionally, the average NU value measures 0.947, and the average D value exceeds 1000. The aforementioned findings demonstrate that the suggested method can successfully enhance the PCNN, improving its capability for image denoising and image segmentation. This can, in part, encourage the use and advancement of the PCNN.

## Introduction

Machine vision technology has advanced significantly in recent decades as a result of the rapid advancement of technology in many industries, including the military, healthcare, and education. The use of machine vision technology has substantially increased the efficacy and efficiency of some jobs as well as the industry’s automation and intelligence^1^. Machine vision technology includes image processing as a significant component, and this technology is the primary means through which machine vision can be made more effective^2,3^. Image segmentation is a key component of image processing technology. Image segmentation is the process of dividing a single image into a number of distinct, non-overlapping regional images using the image’s texture and grayscale distribution area properties to extract the desired target image^4–6^. Researchers have given this technique a lot of attention since it can increase the effectiveness of subsequent image processing as well as the recognition accuracy of the target image. Currently used picture segmentation methods include fuzzy, neural network, and Support Vector Machine methods (SVM)^7,8^. The existing image segmentation algorithms, however, do not achieve the optimal segmentation efficiency and effect, which has an impact on the subsequent image processing and recognition tasks. The pulse-coupled neural network (PCNN) performs better in the area of image segmentation because it possesses the properties of synchronous pulse issuance and spatio-temporal synthesis. The typical PCNN operation procedures, however, are time-consuming, and the output findings are not sufficiently objective. In order to better recover image details while removing noise, an impulse coupled neural network (IWOA PCNN) based on an improved whale optimization algorithm has been proposed^9^. The proposal of this model aims to achieve efficient and highly accurate image segmentation and denoising, which will help to advance the field of machine vision. The research primarily introduces two breakthroughs. The first point is to introduce greedy rules^10^, golden sine ideas^9^, and an improved convergence factor adjustment strategy to optimize WOA^11,12^. This is to improve the optimization performance of WOA and constructing IWOA. The second goal is to improve PCNN’s picture segmentation performance by optimising it using IWOA. The study’s primary substance is divided into four sections. A thorough description and comparison of the pertinent literature and contemporary research findings make up the second section. Building IWOA-PCNN picture segmentation and denoising models is the third section. The performance of the IWOA-PCNN model’s image segmentation and image denoising is examined in the third section. The research is finished in the final section. The study’s innovation is in the use of an enhanced β selection strategy for improving the PCNN image denoising model, thereby accurately identifying image noise points. To achieve global population optimization, the research introduced population information guidance, golden sine, and nonlinear convergence factor adjustment strategies into optimizing the WOA model for local optimization while enhancing the algorithm’s search capability and convergence accuracy.

## Related works

A novel form of artificial neural networks, referred to as PCNN, is currently a subject of lively debate in the field of machine vision. Panigrahy et al. constructed an adaptive two-channel PCNN model with weighted parameters for image fusion and, based on experimental results, suggest that the modified PCNN model may enhance the accuracy and efficiency of image fusion^13^. In order to increase the availability of picture information, Panigrahy et al. developed a parametric adaptive unit, upgraded the two-channel PCNN using it, and then utilised the improved PCNN to fuse infrared light images with visible light images^14^. Experimental findings confirmed the method’s dependability. On the basis of multi-scale morphological gradients, Tan et al. suggested and implemented remote sensing image fusion using an enhanced two-channel PCNN^15^. For all four datasets, the strategy beat current cutting-edge remote sensing image fusion algorithms. Due to the ineffectiveness of current multimodal medical image segmentation algorithms, Wang et al. devised and implemented a PCNN model based on the multi-featured grey wolf algorithm (MFGWO) Optimization^16^. In accordance with the findings, the model performed better than other algorithmic models across the board. In the interest of suggesting a multimodal medical image fusion technique, Nie et al. built a multimodal medical image fusion framework based on PCNN and used the particle swarm algorithm (PSO) to optimise the PCNN parameters^17^. When compared to other picture fusion approaches, the method performs the best. In addition to suggesting an approach for detecting image edges, Deng et al. also improved the emission pattern of PCNN^18^. The outcomes demonstrated the method’s great resilience and efficiency^19^. Lian et al. believe that PCNN has enormous development potential and provides a review of recent research on image segmentation based on PCNN, pointing out the direction for its application and development^20^.

In recent times, machine vision technology has spurred research into image processing techniques. To attain greater accuracy and effectiveness in picture identification, researchers have prioritised image segmentation. It divides original images into predetermined segments, enabling the extraction of intended targets essential to image processing. Li et al. proposed a semi-supervised image segmentation algorithm and applied the algorithm model to the segmentation of medical images^21^. The performance of the method was verified on three medical image segmentation datasets. Isensee et al. proposed a NNU-Net-based medical image segmentation model to address the shortcomings of current medical image segmentation techniques, which are too dependent on the quality of medical image datasets and hardware conditions^22^. The results showed that the model performed relatively well on all 23 public datasets. Chaitanya et al. proposed a medical image segmentation technique based on self-supervised learning and analysed the performance of the technique using three magnetic resonance imaging (MRI) datasets^23^. The results showed that the technique was effective and could meet the practical needs. By combining a microscopic feature clustering strategy, Kim et al. proposed an unsupervised learning-based picture segmentation method. Test trials showed that the technique performed better than numerous other widely used picture segmentation algorithms, proving its viability^24^. Feng et al. used a convolutional neural network (CNN) with an improved network structure to implement medical image segmentation in order to improve the efficiency and accuracy of clinical diagnosis and analysis^25^. The results showed that the improved structure of the CNN was superior in segmenting skin lesion images and retinal lesion images. Yin et al. introduced a depth-guided network to optimise the current U-Net architecture used for medical 2D image segmentation for the drawback that it tends to lead to spatial information loss, resulting in image information loss after image segmentation, to achieve highly accurate medical image segmentation^26^. Sinha et al. introduced a multiscale self-guided attention mechanism into the medical image segmentation domain to improve the image segmentation results^27^. Ibtehaz et al. proposed a MultiResUNet architecture to address the performance shortcomings of the U-Net architecture by modifying its structure^28^. The results showed that the MultiResUNet architecture had an average performance improvement of more than 4% over the U-Net architecture on different datasets.

As mentioned previously, there has been considerable recent research conducted on PCNN and image segmentation in the literature. Additionally, PCNN-based image segmentation approaches have demonstrated success across several domains. It is evident that a single PCNN model’s application effect in the field of image processing is suboptimal, resulting in numerous researchers proposing corresponding strategies to enhance the model’s image processing ability. At present, the optimization path of PCNN mainly utilizes optimization algorithms to obtain the optimal values of important parameters of PCNN, thereby improving the accuracy and efficiency of PCNN models. The current mainstream optimization algorithms, such as Particle Swarm Optimization (PSO), Genetic Algorithm (GA), etc., have not achieved ideal optimization results and efficiency^29–31^. Therefore, a study proposes an IWAO to optimize PCNN, thereby improving the effectiveness of image segmentation and promoting the development of image processing.

## Improved PCNN-based image denoising and segmentation techniques

### Image noise reduction method based on improved simplified PCNN

**Figure 1:**
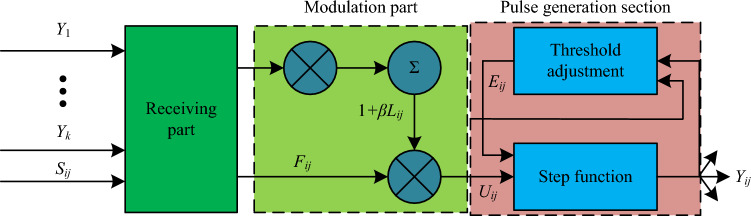
PCNN neuron model.

Pulse coupled neural networks are the third generation of new artificial neural networks, which are composed of three major components: nonlinear connection modulation, dendrites, and pulse generation. The relatively high complexity of unoptimized PCNN structures will lead to a sharp increase in computational complexity. Furthermore, the uneven brightness, high noise, and overall low grayscale values in the original collected images can easily cause significant differences between the greyscale values of the image noise points and the surrounding pixels. In order to accurately extract image noise points, this study proposes a PCNN image denoising model based on an improved $\beta$ selection strategy, which re sets the connection strength and attenuation time constants to rely on the characteristics of the image itself, thus simplifying the number of model parameters. PCNN is a two-dimensional neuronal network with good applications in the field of image segmentation. The PCNN neuronal model is shown in Fig. 1.

**Figure 2:**
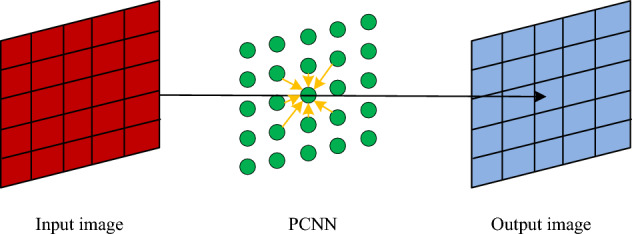
PCNN model.

The receiving portion of the PCNN neuron model in Fig. 1 receives signal information from external sources and from other neurons primarily through two input channels. A connection matrix $M$ and connection weights $W$ link adjacent PCNN neurons to one another. The PCNN model is a single layer of a two-dimensional neuron array composed of many PCNN neuron models connected to each other. In Fig. 2, the PCNN model is displayed.

While processing digital images, PCNN operates on the following concept. When picture data is fed into the PCNN, the brightness data associated with each image pixel point is sent to the appropriate neuron and fed into the A channel. The input of a neuron in the domain is simultaneously coupled to any neuron’s output. When a neuron associated with a pixel sends out a pulse signal, nearby neurons with a comparable grey value to that neuron are activated by the pulse signal and send out their own pulse signal. All neuronal output signals can be classified as either ignited or unignited. $F$ binary picture sequence made up of information on the region, edge, and texture elements of the image will be formed by the sequence of pulses from all neurons. It is possible to do image segmentation, for instance, based on the structure of this sequence. In image processing, the neighbourhood size is typically $3 \times 3$, and Eq. (1) determines the ignition frequency $f_{ij}$ of any neuron.

1 $$f_{ij} = \frac{{\alpha_{E} }}{{\ln \left( {1 + \frac{{V_{E} }}{{U_{ij} }}} \right)}}.$$

**Figure 3:**
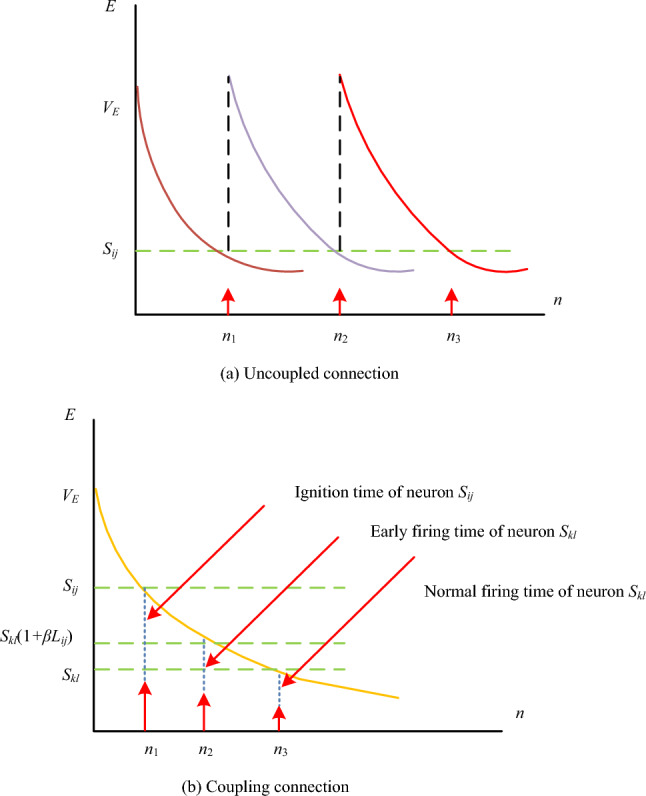
Ignition of neurons.

$\alpha_{E}$ is the attenuation coefficient of the pulse production section’s threshold in Eq. (1). The pulse generation section’s threshold’s amplitude coefficient is represented by the letter $V_{E}$. After the modulation portion, $U_{ij}$ represents the neuron’s internal activity term. It is evident that the neuron’s firing frequency and pixel point brightness are inversely correlated. Figure 3 depicts the two types of neuron ignition: coupled connection and uncoupled connection.

Figure 3a depicts neuron firing in the absence of coupling connections. In this state, each neuron lacks connections and operates independently of others. The connection strength between adjacent neurons is equal to 0. On the other hand, Fig. 3b illustrates neuron firing under coupled connections. In this state, neurons are interconnected and mutually influence each other, and the connection strength between adjacent neurons is non-zero. When the grey scale values of the noisy pixel points differ significantly from those of the normal image pixel points, the PCNN modifies the luminance of the pixel points and fields to identify and denoise the noisy pixel points. However, because PCNN’s structure is more intricate, it requires more computing while processing images, which has an impact on how effective it is. A simpler PCNN model is suggested in the study as a solution to this issue. Initially, Eq. (2) is used to express the neuron’s input term.

2 $$F_{ij} \left( n \right) = I_{ij} .$$

In Eq. (2), $I_{ij}$ is the external input excitation signal, i.e. the grey value of the pixel point with coordinates $\left( {i,j} \right)$ in the image. $F_{ij} \left( n \right)$ denotes the input term of the pixel point with coordinates $\left( {i,j} \right)$ in the $n$th neuron. The connected input to the neuron $L_{ij}$ is represented as Eq. (3).

3 $$L_{ij} \left( n \right) = \sum\limits_{k,l} {W_{ijkl} Y_{kl} \left( {n - 1} \right)} .$$

In Eq. (3), $\left( {k,l} \right)$ is the coordinate of another pixel point; $W_{ijkl}$ is the coupling connection matrix between pixel points; and $Y_{kl}$ is the pulse output of the neuron corresponding to the pixel point with coordinate $\left( {k,l} \right)$. The internal activity term $U_{ij}$ of the neuron is expressed as Eq. (4).

4 $$U_{ij} \left( n \right) = F_{ij} \left( n \right)\left( {1 + \beta L_{ij} \left( n \right)} \right).$$

In Eq. (4), $\beta$ is the strength of the connection between adjacent neurons and is one of the important parameters affecting the performance of the PCNN model. The mathematical expression for the pulse output $Y_{ij}$ is shown in Eq. (5).

5 $$Y_{ij} \left( n \right) = \left\{ {\begin{array}{*{20}c} {1,\;U_{ij} \left( n \right) > E_{ij} \left( n \right)} \\ {0,\;U_{ij} \left( n \right) \le E_{ij} \left( n \right)} \\ \end{array} } \right..$$

In Eq. (5), $E_{ij}$ is the dynamic threshold of the neuron, which is calculated by Eq. (6).

6 $$E_{ij} \left( n \right) = \exp \left( { - \alpha_{E} } \right)E_{ij} \left( {n - 1} \right) + v_{E} Y_{ij} \left( n \right).$$

In Eq. (6), $\alpha_{E}$ is the decay time constant of the dynamic threshold and $v_{E}$ is the amplification factor of the threshold output. The input and connection domains in the traditional PCNN model are simplified by Eqs. (2) to (6), and thus a simplified PCNN model is constructed. In the simplified PCNN model, the parameter values of $\beta$ for the connection strength between adjacent neurons are manually adjusted by multiple experiments. This takes more time and affects the image denoising efficiency of the PCNN model. For this reason, the study proposes an improved $\beta$-value selection strategy. This approach selects a 3 × 3 neighborhood with the pixel as the centre, sorts the grayscale values of the pixels, calculates the median of the neighborhood, subtracts the median value from the grayscale value of the pixel and then divides the result by the maximum grayscale level of the pixel. When a significant disparity in grayscale values exists, the greater the connection strength, the higher the internal activity, encouraging the capacity for coordinated ignition and successful exclusion of noise points. Hence, only noise points undergo subsequent processing. First, the $J_{ij}$ is used to represent the grey value of the pixel, and the median of the $3 \times 3$-matrix ordering composed of neurons adjacent to the $J_{ij}$ is represented as $J_{kk}$. At this point, the $\beta$ is calculated using Eq. (7).

7 $$\beta_{ij} = \left\{ {\begin{array}{*{20}c} {\left( {J_{ij} - J_{kk} } \right)/255} & {\left( {J_{ij} \ne J_{kk} } \right)} \\ 0 & {\left( {J_{ij} = J_{kk} } \right)} \\ \end{array} } \right..$$

The highest value of the pixel grey level in Eq. (7) is 255. On the basis of the aforementioned, PCNN enhancement is finished. The image denoising efficiency and denoising effect can be successfully increased when employing the upgraded PCNN. The impact of image denoising is assessed using the peak signal-to-noise ratio (PSNR). A higher PSNR value means that the image’s noise signal is less, which suggests that the image denoising process is more effective.

### IWOA-PCNN based image segmentation model

**Figure 4:**
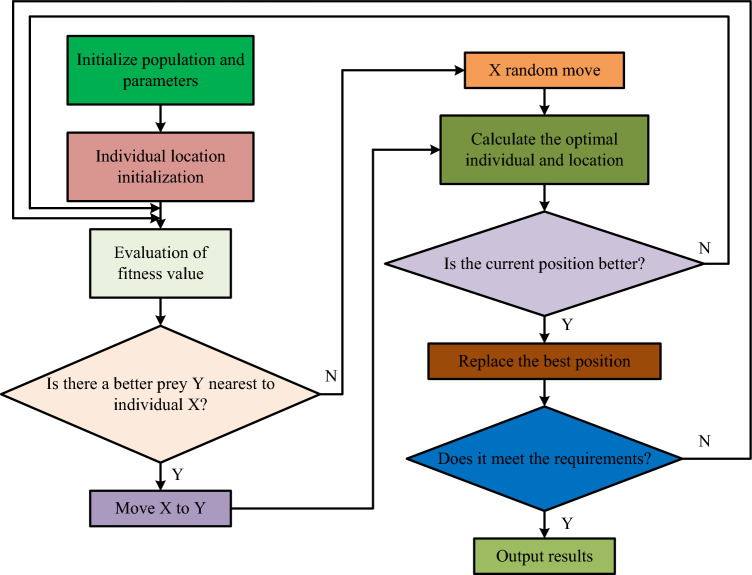
WOA optimization process.

Considering that the parameter selection has a significant impact on the image segmentation performance, and generally using empirical methods or manual debugging for parameter selection will result in a large amount of manpower consumption. To improve efficiency, this study introduces the WOA algorithm to perform intelligent parameter optimization. Pixel grey values are also the basis for PCNN-based image segmentation. The goal of image segmentation is to identify and separate the pixels in an image that have a grey value comparable to the target region that the user is trying to identify. The idea behind PCNN-based image segmentation is that neurons with identical luminance values next to high brightness pixels in the image would produce impulse signals in parallel as a matter of priority. The segmentation of an image is achieved by a similar mapping of PCNN neurons. In image segmentation tasks, the performance of PCNN models is often influenced by their parameters. In the above, the study simplifies the PCNN model and proposes an improved $\beta$-value selection strategy. However, in addition to the $\beta$-value, the decay time constant $\alpha_{E}$ of the dynamic threshold and the amplification factor $v_{E}$ of the threshold output also have a direct impact on the performance of the PCNN model. In the orthodox approach, researchers select the optimal values of these two parameters through multiple manual experiments, necessitating a considerable investment of time and effort. However, these experimental outcomes may not be entirely objective, leading to suboptimal PCNN model performance and adversely affecting image segmentation results. To address the above problems, the study uses WOA to obtain the optimal $\alpha_{E}$ and $v_{E}$ values of the model based on a simplified PCNN model, so as to optimize the PCNN model. The Optimization process of WOA is shown in Fig. 4.

In the process shown in Fig. 4, the information on the optimal feeding position of an individual whale is the optimal $\alpha_{E}$ and $v_{E}$ values. In the WOA, the distance $D$ between an individual and the current optimal individual at the $t$th iteration can be expressed as Eq. (8).

8 $$D = \left| {CX^{*} \left( t \right) - X\left( t \right)} \right|.$$

In Eq. (8), $X$ denotes the whale individual position; $X^{*}$ denotes the optimal individual position at the $t_{th}$ iteration, and $C$ is the coefficient vector. According to Eq. (8), the position of the individual whale at the $t + 1$ th iteration can be obtained, as in Eq. (9).

9 $$X\left( {t + 1} \right) = X^{*} \left( t \right) - AD.$$

$A$ is the coefficient vector in Eq. (9). In the WOA algorithm, the individual with the smallest population fitness value is designated as the current optimal individual, and the generation of the global optimal position is reliant on the current optimal individual of the whale population following multiple iterations. While the overall process ensures that the population remains close to the prey position, it disregards the impact of other individuals on the global optimal solution, making it susceptible to escaping from local extremes. Therefore, to enhance the accuracy of whale population optimization, this study suggests a strategy guided by population information to enhance the WOA algorithm, as indicated by formula (10).

10 $$X^{*} \left( t \right) = w_{3} X_{1} \left( t \right) + w_{2} X_{2} \left( t \right) + w_{1} X_{3} \left( t \right).$$

Since the number of iterations is $t$, position $X_{1} \left( t \right),X_{2} \left( t \right),X_{3} \left( t \right)$ in Eq. (10), which is the best position other than $X_{1} \left( t \right)$ and has the lowest fitness value, is the best place in the whale population. The weight of $X_{1} \left( t \right),X_{2} \left( t \right),X_{3} \left( t \right)$ is given by $w_{1} ,w_{2} ,w_{3}$. $X_{1} \left( t \right),X_{2} \left( t \right),X_{3} \left( t \right)$ and its associated weight $w_{1} ,w_{2} ,w_{3}$ are not matched one to one in Eq. (10), but rather as an inverse ratio. The best individual, the second-best individual, and the better individual can share knowledge more effectively and have a greater impact on the overall optimal position by using Eq. (10). The aforementioned techniques can successfully prevent WOA early convergence. Based on the foregoing, the study adds greedy principles to assess how superior the whale individuals’ current position is compared to their prior position and to hold onto the better position. The whale hunting process in the conventional WOA algorithm is based on the best positional data and random coefficients throughout this iteration, however this approach will disregard some individuals in the algorithm and have an impact on the precision of the superiority search. The greedy rule refers to always selecting the current optimal solution when solving a problem. Therefore, the solution obtained under the greedy rule is often a local optimal solution. The greedy rule generally divides the solving steps of a problem into four steps: first, mathematical modeling of the problem to be solved; secondly, decompose the problem to obtain multiple sub problems; then, solve these decomposed sub problems to obtain local optimal solutions for all sub problems; finally, merge all the sub problem solving results and solve the problem to be solved. In the research proposed, greedy rules were used to compare the new and original positions of whale individuals, preserving the dominant individuals and ensuring the accuracy of WOA solution.

Individuals in the WOA algorithm do not consider the central position of the population or the previous generation’s position when pursuing their prey. They rely solely on the current optimal position and associated random coefficients. Consequently, the whale population cannot fully cover the entire search space, and certain possible target positions become ignored, thereby diminishing the algorithm’s accuracy. Random coefficients can compromise the stability of the algorithm during the optimization process. To address this challenge, an improved golden sine algorithm is adopted after the random search phase of the prey to accelerate the optimization speed of the algorithm, expand the search area of the whale population in the solution space, and thereby improve the global search ability and convergence accuracy of the algorithm. The Golden Sine Algorithm is a new heuristic algorithm proposed in recent years, based on the sine function. Its principle is to traverse all values on the sine function during position updates, introducing the golden section number to reduce the solution space so that the algorithm only traverses the region that produces the optimal solution, thereby improving the efficiency and accuracy of the algorithm’s problem solving. In response to the above issues, the golden sine idea is introduced to improve the hunting process of WOA, as shown in Eq. (11).

11 $$\begin{aligned} X\left( {t + 1} \right) = & X\left( t \right) \cdot \left| {\sin \left( {R_{1} } \right)} \right| \\ & + R_{2} \cdot \sin \left( {R_{1} } \right) \cdot \left| {x_{1} \cdot X^{*} \left( t \right) - x_{2} X_{mean} \left( t \right)} \right|. \\ \end{aligned}$$

In Eq. (11), $X_{mean} \left( t \right)$ is the average position of all individuals in this iteration; $R_{1}$ and $R_{2}$ are random numbers, which are mainly responsible for regulating the distance and direction of individuals’ movement, and $x_{1} ,x_{2}$ is the golden mean coefficient, which mainly serves to guide individuals to move towards the location of the best prey. The WOA algorithm’s capacity to search for global superiority can be accelerated via a strategy based on Eq. (11). To control the convergence and global search capabilities of the algorithm, WOA frequently inserts a convergence factor $a$ during the iterative phase. The WOA algorithm’s convergence factor, represented by ‘a’, diminishes linearly from 2 to 0 with the increase of iterations, ultimately leading to a decrease in the algorithm’s ability to explore globally. This causes an inadequate search in the initial stages and slower iteration speed in the later stages. The algorithm’s actual search process cannot be better reflected by the linear changes in parameter ‘a’. However, in swarm intelligence optimization algorithms that rely on population iteration, it is imperative to achieve a balance between the algorithm’s capacity for global search and its ability to encourage local development. Control factors play an integral role in the evolutionary process. Furthermore, parameter A’s value alters according to the convergence factor a. Greater values of convergence factor can improve the algorithm’s ability to explore globally, preventing it from being limited to local optima, whereas smaller values enhance its capacity to develop locally and quicken the algorithm’s convergence speed. To enhance the influence of convergence factors on algorithm performance, a nonlinear convergence factor adjustment strategy is proposed without changing the trend of the original convergence factor, as shown in Eq. (12).

12 $$a = \left\{ {\begin{array}{*{20}l} {\frac{{T_{\max }^{2} }}{4}\left( {\left( {t - \frac{{T_{\max } }}{2}} \right)^{2} + 1} \right),} & {t \le \frac{{T_{\max } }}{2}} \\ {\frac{{T_{\max }^{2} }}{4}\left( {t - \frac{{T_{\max } }}{2}} \right)^{2} ,} & {t > \frac{{T_{\max } }}{2}} \\ \end{array} } \right..$$

**Figure 5:**
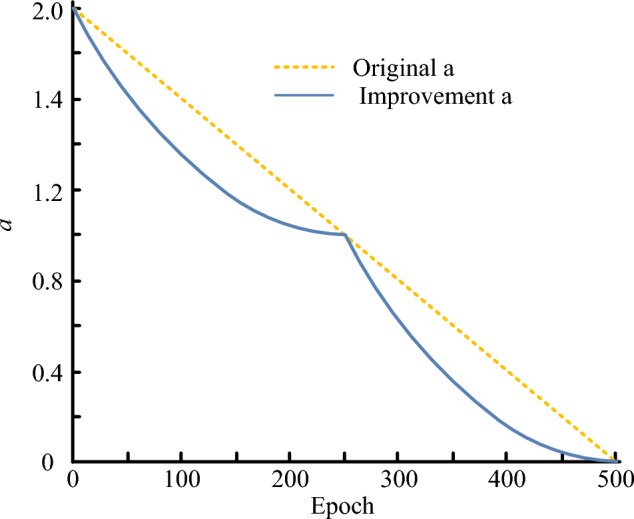
The change trend between the improved convergence factor adjustment strategy and the original convergence factor adjustment strategy.

$T_{\max }$ is a predetermined upper limit of the iteration in Eq. (12). In hopes of ensuring the global optimization-seeking performance of the WOA algorithm in the early iterations and convergence in the late iterations, non-linear and segmental changes can be made using the convergence factor adjustment technique illustrated in Eq. (12). Figure 5 displays the trend between the enhanced convergence factor adjustment approach and the original convergence factor adjustment strategy.

Depending on the foregoing, the IWO algorithm model can be built, and the multi-strategy collaborative improvement of WOA can be finished to enhance its optimization performance. The IWOA-PCNN model is created to enhance the performance of the PCNN model in image segmentation and image denoising. IWOA is used to discover the best parameters for simplifying the PCNN model to improve its performance.

## Image denoising and segmentation performance verification of IWOA-PCNN model

### Image denoising performance analysis of IWOA-PCNN model

**Figure 6:**
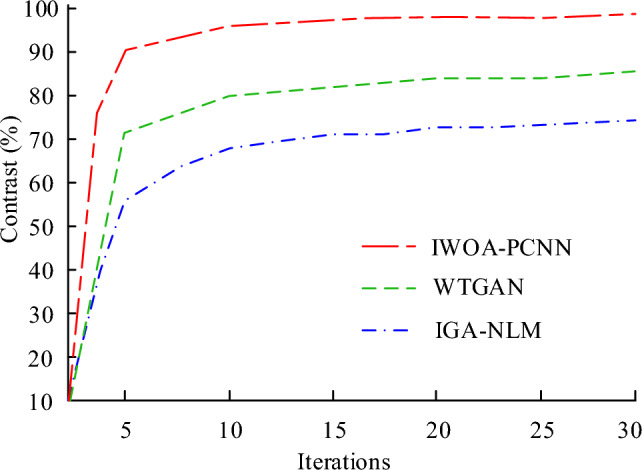
Image contrast curves of different models.

The work streamlines the PCNN model in order to enhance its performance in image denoising and image segmentation. And in order to create the IWOA-PCNN model, the IWOA algorithm is employed to discover the optimisation of the key PCNN model parameters. The study designed tests with the following experimental environment in order to validate the functionality of the IWOA-PCNN model. The experimental platform is MATLAB 2016a software, running on Windows 7 with an Intel i3 processor and 4 G of memory. The experimental dataset came from an online repository. Secondly, the IWOA-PCNN model’s image denoising performance is confirmed. There are two cutting-edge image denoising algorithm models: the enhanced genetic algorithm optimised non-local mean image denoising algorithm, and the denoising model integrating wavelet transform and adversarial generative network (WTGAN) (IGA-NLM)^32–34^. In terms of performance indicators for image enhancement models, Peak Signal to Noise Ratio (PSNR) is an engineering term that represents the ratio of the maximum possible power of a signal to the destructive noise power that affects its representational accuracy. Because many signals have a very wide dynamic range, Peak Signal to Noise Ratio (PSNR) is usually expressed in logarithmic decibel units^35,36^. Structural Similarity (SSIM) is an indicator that measures the similarity between two images. Regional Uniformity (NU) refers to the uniformity and consistency of features within the segmented region^16^. The consistency indicators within the region are mainly based on information such as grey scale, colour, texture and entropy of the image. Inter-class difference refers to the degree of difference in pixel values between different classes. F1 score is defined as the harmonic mean of the precision and recall rates. Recall measures the recall rate of the retrieval system. Area Under Curve (AUC) is defined as the area enclosed by the coordinate axis under the receiver operating characteristic curve^37^. Figure 6 compares the capacities of the three aforementioned algorithms for image denoising. The IWOA-PCNN model has the best image denoising performance, as seen in Fig. 6, producing a cleaner image with higher contrast.

**Figure 7:**
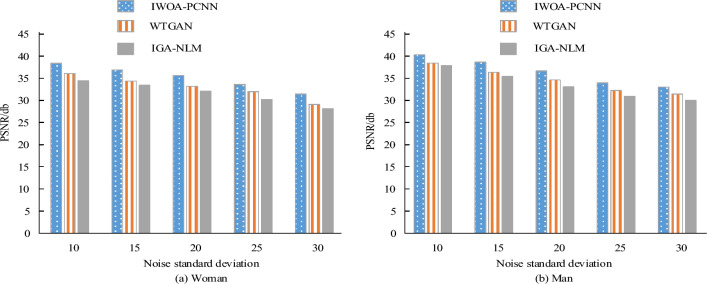
PSNR under different noise standards.

A four-part additive Gaussian white noise with noise standard deviations of 10, 15, 20, 25, and 30 was added to the experimental data set (see Fig. 7). Table 1 compares the PSNRs of the various noise-filled images following processing with the three aforementioned techniques. The average PSNR of each individual picture following IWOA-PCNN processing is 35.87, which is 2.09 and 3.31 higher than WTGAN and IGA-NLM, respectively, as shown in Table 1.

**Table 1:** MSE under different noise standards.

Image	Noise standard deviation	MSE		
		IWOA-PCNN	WTGAN	IGA-NLM
Woman	10	0.06	0.09	0.12
	15	0.14	0.17	0.22
	20	0.25	0.31	0.35
	25	0.31	0.37	0.40
	30	0.45	0.52	0.57
Man	10	0.07	0.13	0.15
	15	0.14	0.19	0.23
	20	0.21	0.25	0.29
	25	0.32	0.38	0.44
	30	0.41	0.53	0.59
Average	–	0.24	0.29	0.34

The MSEs of the different noisy images after comparing the processing of the three algorithms mentioned above are shown in Table 1. In Table 1, it can be observed that the average MSE of each image after IWOA-PCNN processing is 0.24, which is 0.05 and 0.10 lower than WTGAN and IGA-NLM respectively.

**Figure 8:**
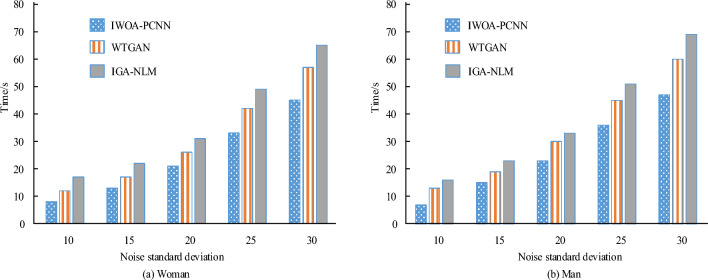
The time required for three algorithms to process different noisy images.

Figure 8 compares the processing time of three algorithms for numerous images containing noise. Table 1 illustrates that IWOA-PCNN requires 24.80 s to process several noisy images. This result is 7.30 and 7.76 s quicker than WTGAN and IGA-NLM, respectively. Consequently, the study recommends IWOA-PCNN as it exhibits superior denoising efficiency and effect compared to existing denoising methods.

**Figure 9:**
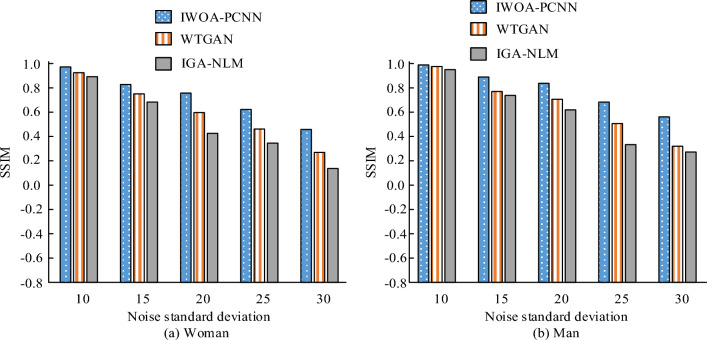
SSIM of several algorithm models.

Evaluate the image denoising effect of several algorithm models using SSIM indicators, as shown in Fig. 9. In Fig. 9a, it can be seen that in the image “woman”, when the noise standard deviation is 10, the SSIM value of the IWOA-PCNN model is very close to 1, which is 0.986. This indicates that after using the IWOA-PCNN model to denoise the image, the denoised image obtained is very similar to the original image, thus verifying the image denoising effect of the IWOA-PCNN model. The SSIM values of the WTGAN model and the IGA-NLM model are 0.963 and 0.925 respectively, which are lower than those of the IWOA-PCNN model. This indicates that when the noise standard deviation is 10, the denoising effects of the three algorithm models are relatively good, but the IWOA-PCNN model has the best image denoising effect. When the noise standard deviation is 15, the SSIM value of the IWOA-PCNN model is 0.842, which is higher than that of the WTGAN model and IGA-NLM model. When the noise standard deviation is 20, 25 and 30, the SSIM values of the IWOA-PCNN model are 0.793, 0.652 and 0.467 respectively, which are higher than those of the WTGAN model and the IGA-NLM model. It is clear from the preceding discussion that an increase in the noise content results in a decrease in the similarity between the image processed by an image denoising algorithm model and the original image. Nonetheless, for varying levels of noise, applying the IWOA-PCNN model improves the SSIM values between the processed and original images more so than the WTGAN and IGA-NLM models. Figure 9b shows that when the standard deviation of noise is 10, 15, 20, 25, and 30, the SSIM values between the obtained image and the original image are higher with the IWOA-PCNN model compared to the WTGAN model and the IGA-NLM model. This trend is consistent with the results presented in Fig. 9a. These findings suggest that the IWOA-PCNN model is more effective than the WTGAN model and the IGA-NLM model in image denoising across various scenarios.

### Performance analysis of IWOA-PCNN model for image segmentation

**Figure 10:**
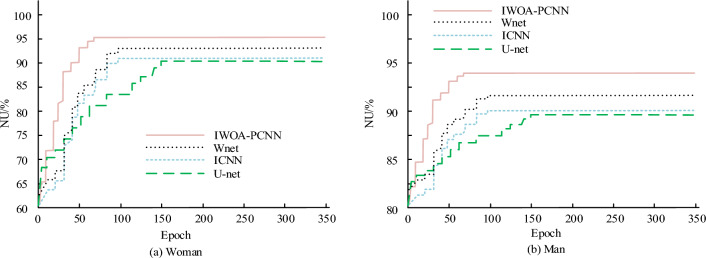
NU value processed by three algorithms.

The study simplifies the PCNN model and optimises it using IWOA to obtain the IOWA-PCNN model, and applies it to image segmentation. The two more advanced image segmentation algorithms currently available are the ICNN model and the U-Net model^38,39^. The image segmentation performance of the three algorithms is compared to verify the image segmentation effect of the IWOA-PCNN model. Region uniformity (NU) assessed the efficacy of image segmentation. Figure 10 indicates NU values following deployment of three algorithms. Specifically, in Fig. 10a, the IWOA-PCNN model produced a NU value of 0.952, which is 0.036 and 0.040 larger than that of the ICNN model and the U-Net model respectively. In Fig. 10b, the NU value of the IWOA-PCNN model is 0.941, which is 0.032 and 0.035 higher than that of the ICNN model and the U-Net model respectively. For the different images, the average NU value of the IWOA-PCNN model was 0.947, which was 0.034 and 0.038 higher than that of the ICNN model and the U-Net model, respectively. Figure 10 shows that the model suggested in the study outperformed the WNet model in terms of NU metrics on both datasets by more than 2%.

**Figure 11:**
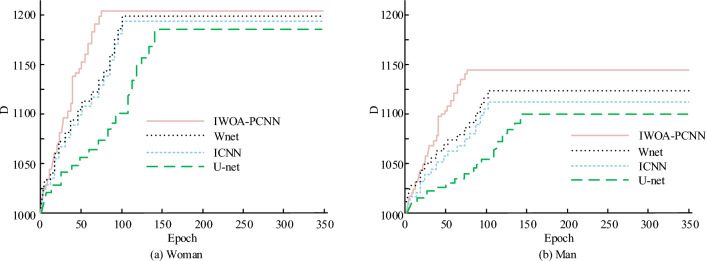
D value processed by three algorithms.

The inter-class variance (D) was used to evaluate the effectiveness of image segmentation. Figure 11 illustrates the D values for the three techniques in various photos. The D-value of the IWOA-PCNN model in Fig. 11a is 1221.30, surpassing those of the ICNN model and U-Net model by 10.60 and 15.30, respectively. The IWOA-PCNN model in Fig. 11b exhibits a D-value of 1146.50, which is 28.40 and 34.60 greater than the D-values of the ICNN and U-Net models, correspondingly. The IWOA-PCNN model has an average D-value of 1183.90, which is 12.95 and 24.95 greater than the ICNN model and the U-Net model, respectively, as can be seen from the two photos. From Fig. 11, the model proposed in the study outperforms the WNet model in terms of inter class difference indicators by more than 10.

**Figure 12:**
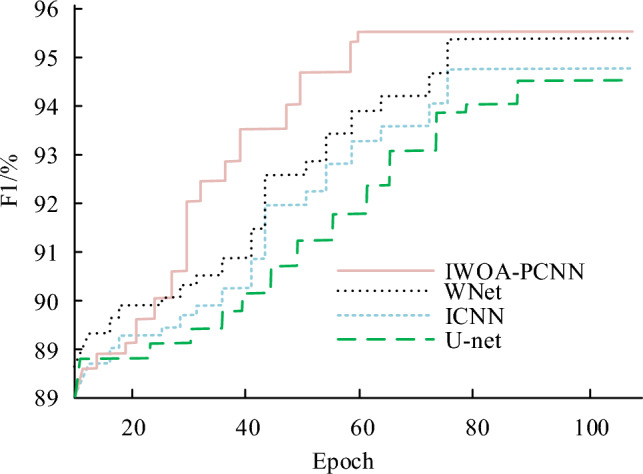
F1 value processed by three algorithms.

Compare the F1 values of different algorithm models as shown in Fig. 12. It can be seen that as the number of iterations of the algorithm increases, the F1 values of the IWOA-PCNN model, ICNN model and U-Net model continue to increase. When the F1 value increases to a certain extent, the F1 values of the IWOA-PCNN model, ICNN model and U-Net model stop increasing and the curve basically does not change. The performance of the algorithm model has now been refined to the best possible level. After 40 iterations, the F1 values of the three algorithm models are already significantly different from each other, and the F1 values of the IWOA-PCNN model are significantly higher than those of the ICNN model and the U-Net model. After reaching the optimal state, the F1 value of the IWOA-PCNN model reached 95.64%, which is 1.45% and 1.62% higher than the ICNN and U-Net models, respectively.

**Figure 13:**
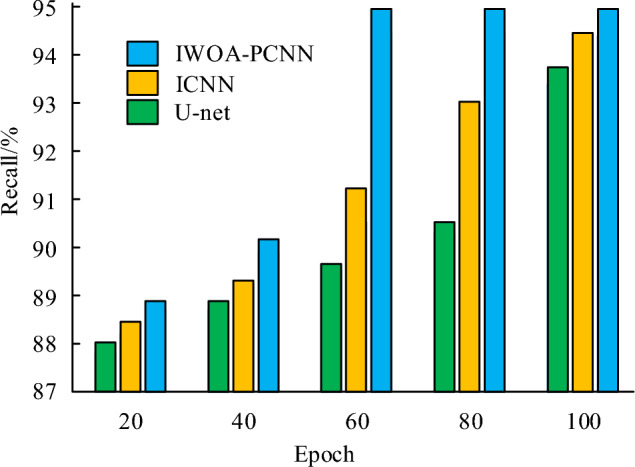
Recall value processed by three algorithms.

Compare the Recall values of several algorithm models, as shown in Fig. 13. It can be seen that the trend of F1 value change is similar to that of the model. As the number of iterations of the algorithm increases, the Recall values of the IWOA-PCNN model, ICNN model, and U-Net model all continuously increase. When the Recall value increases to a certain extent, the Recall values of the IWOA-PCNN model, ICNN model, and U-Net model no longer increase, and the curve basically does not change. The IWOA-PCNN model requires significantly fewer iterations to reach its optimal state than the ICNN model and U-Net model. It can be seen that when the number of iterations reaches 60, there is already a significant difference in the Recall values of the three algorithm models, and the Recall values of the IWOA-PCNN model are significantly higher than those of the ICNN model and U-Net model. After reaching the optimal state, the Recall value of the IWOA-PCNN model reached 95.00%, both higher than the CNN model and U-Net model. The above results can indicate that the convergence and image segmentation performance of the IWOA-PCNN model are significantly better than those of the ICNN model and U-Net model.

**Figure 14:**
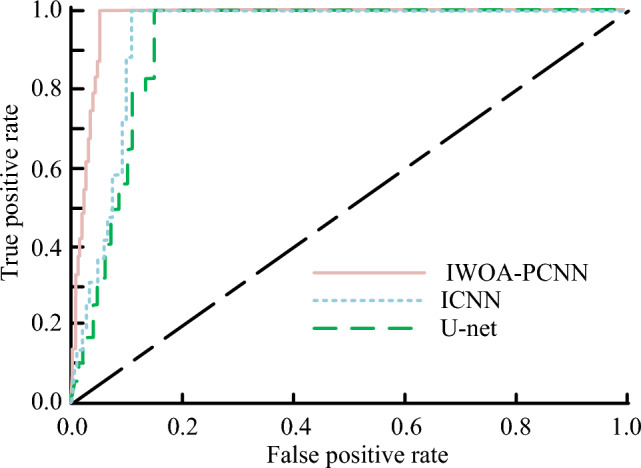
Comprehensive performance of three algorithms for image segmentation.

As shown in Fig. 14, the AUC value of the IWOA-PCNN model is 0.994, which is 0.008 and 0.014 higher than that of the ICNN model and the U-Net model correspondingly. The suggested IWOA-PCNN model performs well in both image denoising and image segmentation tasks and is able to complete these tasks quickly and accurately, which advances the field of image processing technology.

**Figure 15:**
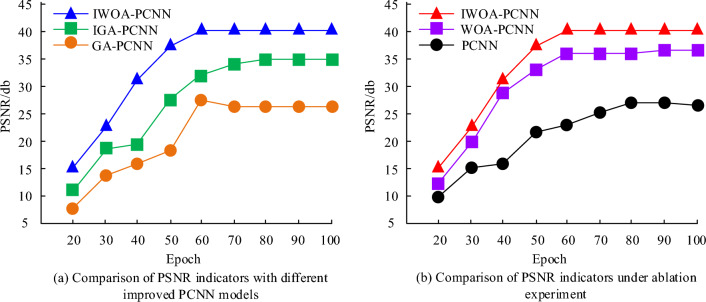
The comprehensiveness of three image segmentation algorithms.

For various experiments comparing the performance of the improved PCNN and for ablation experiments, 100 images from BSD400 and 100 images from the Waterloo exploration database were selected for model validation. Due to the different regions of the image containing different detail textures, in this article, each image is cropped into blocks of size 35 × 35. From Fig. 15a, in terms of PSNR indicators, the IWOA-PCNN model proposed by the Research Institute finally achieved a convergence value of 40 db, while the IGA-PCNN and GA-PCNN models achieved convergence values of 35 db and 26.7 db, respectively. The comparison shows that the proposed model improves the PSNR indicators by 5 db and 13.3 db compared to the latter two models. Additionally, Fig. 15b illustrates that during the ablation experiment, both the unoptimized PCNN model and the optimized PCNN model through unmodified WOA achieved PSNR convergence values lower than 36. On the other hand, the proposed model in this study attained a PSNR convergence value of 40, indicating its superiority in image denoising and optimization.

**Figure 16:**
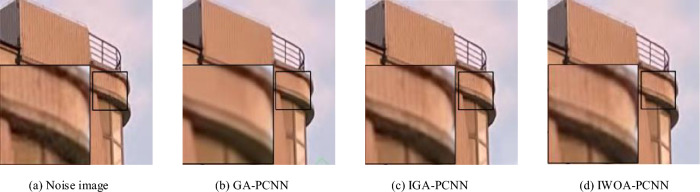
Comparison of denoising effects on real captured images.

From the analysis of denoised images captured in reality, as shown in Fig. 16, it is evident that the GA-PCNN model generates artifacts and flaws in the denoised images, leaving behind residual noise. After processing noisy images, the IGA-PCNN model blurs the details, leading to partial defects and artifacts at the edges of the object. In contrast, the research institute’s proposed model achieves good denoising results by removing noise as much as possible while preserving clear edge details. This is accomplished without harming the texture structure, engaging in excessive smoothing, or generating artifacts.

## Conclusion

PCNNs are frequently utilised in image denoising and image segmentation tasks. The efficiency and effectiveness of these tasks are unsatisfactory due to certain imperfections within the current PCNN model. Consequently, an IWOA-PCNN model is suggested to rectify these shortcomings and enhance the model’s functionality and effectiveness. According to the results, the IWOA-PCNN model had the best image denoising performance, producing cleaner images with more preserved features. The results showed that the image segmentation and denoising model proposed in the study had higher values in indicators such as PSNR and inter class differences, indicating that the model has superior denoising and image quality improvement capabilities in image optimization. The suggested IWOA-PCNN model can segment and denoise images quickly and accurately, which has a catalytic influence on the advancement of image processing technology. The study uses the IWOA algorithm in conjunction with the PCNN model to increase the accuracy of the PCNN model. IWOA needs to be further optimized in subsequent research in the hope of reducing the complexity of the model, as this strategy increases the complexity of the overall algorithm model and the computational effort required to run it. However, this method will lead to an increase in the complexity and computational complexity of the overall algorithm model. In addition, the application effectiveness of this model in practical scenarios has not been verified. In future research, further optimization of the IWOA algorithm is needed to reduce the complexity of the model and verify its practical application effectiveness. By further optimizing and improving the model and verifying its practicality, the image processing performance of the model in different scenarios can be obtained, providing guidance for practical application and further improvement of the model. Automating the setting of PCNN model parameters is still a challenge despite the direct substitution of the optimal threshold for image segmentation reducing the need for time decay parameter arrangement. However, the connection coefficient still requires manual configuration.

## Data Availability

The data used to support the findings of this study are all in the manuscript.
